# Histomorphological and Immunohistochemical Subtyping of Ampullary Adenocarcinoma and Its Association With Survival Status: An Ambispective Observational Study

**DOI:** 10.7759/cureus.114282

**Published:** 2026-08-10

**Authors:** Namisha Goyal, Ranjana Giri, Prita Pradhan, Subrat Sahu

**Affiliations:** 1 Pathology, Kalinga Institute of Medical Sciences, Bhubaneswar, IND; 2 Surgery, Kalinga Institute of Medical Sciences, Bhubaneswar, IND

**Keywords:** cdx2, ck20, ck7, immunomorphological subtype (ims), intestinal, pancreatobiliary, periampullary carcinoma

## Abstract

Background: Periampullary adenocarcinoma (PAC) is a rare and unique malignancy arising from structures around the ampulla. Based on the histomorphology, two common subtypes include pancreatobiliary (PB) and intestinal (INT), which have therapeutic and prognostic significance. There are limited studies that highlighted the magnitude of interobserver variability in subtyping. The aims of the study were primarily to assess the morphologic and IHC expression of CK7, CK20, and CDX2 to differentiate between pancreatobiliary and intestinal types of PAC and secondarily to compare the subtype with pathological grade, stage, lymph node status, lymphovascular invasion, and survival status.

Materials and methods: This was an ambispective study of six years' duration, including prospective cases from June 2022 to May 2024 (two years) and retrospective cases from January 2018 to May 2022 (four years) in the Department of Pathology, Kalinga Institute of Medical Sciences (KIMS) and Pradyumna Bal Memorial Hospital (PBMH), Bhubaneswar. All pancreaticoduodenectomy specimens received for adenocarcinoma were included in the study. The histomorphologic features were studied and documented. The cases were sub-classified as PB and INT based on standard criteria. Discrepant cases were reviewed for consensus morphology (CM). CK7 was used as a marker for PB type, while CK20 and CDX2 were used for INT type. The CM and immunomorphologic subtype (IMS) were studied and documented. Statistical tools like correlation analysis and comparative analysis were used in the study.

Results: A total of 33 pancreaticoduodenectomy specimens were received during this period. Our study comprised subjects belonging to 32 to 74 years with a mean age of 55.9 years, including 19 males (57.6%) and 14 females (42.4%). Histomorphological subtyping was done by two different observers as per the criteria, which showed slight agreement in architecture (k = 0.2), desmoplastic stroma (k = 0.1), luminal necrosis (k = 0.2), and presence of goblet cells (k = 0.1); however, no agreement was observed in cytomorphology (k = -0.2) and nuclear features (k = -0.2). Interobserver variation was noted in 51.5% cases (17/33) with no agreement between the observers (k = 0.1, p = 0.6). IMS showed a significant moderate degree of agreement with the diagnosis made on CM (k = 0.6, p ≤ 0.001).

Conclusion: With optimal sampling, the use of CM along with adjunct immunohistochemistry to recognize the IMS, accurate classification of PAC is possible in most cases, which has therapeutic and prognostic significance.

## Introduction

Periampullary adenocarcinoma (PAC) is a unique malignancy that collectively encompasses primary adenocarcinomas arising from structures around the ampulla. It includes malignancies of the head of the pancreas, distal bile duct, and duodenum. It is an uncommon tumor with an incidence of 0.5-2% of all gastrointestinal malignancies [1]. It stands apart owing to the anatomical complexity of the location, where there is a confluence of both pancreatobiliary and intestinal epithelium at the papilla of Vater [2]. Based on the histomorphology, two common subtypes of PACs can be recognized, namely, "pancreatobiliary," which resembles pancreatic duct epithelium, and "intestinal," which resembles the colonic type of intestinal epithelium [3]. The former likely takes origin from pancreatic intraepithelial neoplasia, while the latter develops from intestinal mucosae following the adenoma-carcinoma sequence [2]. 

The subtyping of these tumors is important both from the therapeutic and prognostic point of view, but they pose diagnostic dilemmas due to morphologic overlap [4,5]. There are limited studies that highlighted the magnitude of interobserver variability by using histomorphological criteria, which are based on cytonuclear and architectural parameters. This study aimed to highlight the discrepancies in interobserver variations in the histomorphology-based subtyping in the uncommon group of periampullary tumors. The primary objective was to assess the morphologic and IHC expression of CK7, CK20, and CDX2 to differentiate between pancreatobiliary and intestinal types of PAC, and the secondary objective was to compare the subtype with pathological grade, stage, lymph node status, and lymphovascular invasion.

## Materials and methods

Study design and study setting

This was an ambispective study of six years' duration, including prospective cases from June 2022 to May 2024 (two years) and retrospective cases from January 2018 to May 2022 (four years) in the Department of Pathology, Kalinga Institute of Medical Sciences (KIMS) and Pradyumna Bal Memorial Hospital (PBMH), Bhubaneswar. This study was approved by the Institutional Ethics Committee (IEC) with approval no. KIIT/KIMS/IEC/943/2022 and was performed in accordance with the ethical principles laid down in the Declaration of Helsinki 1975, as revised in 2013.

Sample size calculation

Consecutive cases of pancreaticoduodenectomy specimens meeting the inclusion and exclusion criteria were taken in the study. Based on the incidence, the target sample size was 33.

Study population

All pancreaticoduodenectomy specimens as available in the pathology department (collected during January 2018 to May 2024) were included, while small biopsies, tissue unsatisfactory for IHC, and recurrent or repeat cases were excluded from the study. Retrospective data from the records and paraffin blocks from the archives were retrieved. With consent, the age, sex, clinical presentation, site of tumor, size of tumor, radiological picture, and type of surgery were noted.

All pancreaticoduodenectomy specimens with tumor at the ampullary region, as identified both radiologically and on gross examination, were sectioned and sampled as per the standard College of American Pathologists (CAP) protocol along with margins and lymph nodes [6]. Samples were processed as per the standard operating procedures (SOP) of the laboratory in the tissue processor (Leica TP 1020 Histokinette). Sections were cut and stained with hematoxylin and eosin (H&E) as per the SOP of the laboratory. For survival analysis, data were collected from each patient prospectively.

Histomorphological findings

The H&E-stained slides were reviewed by two independent pathologists (Observers 1 and 2) who were blinded to the final diagnosis and IHC. As the morphology of PAC is very confusing due to its location and has a high chance of interobserver variation, two independent pathologists were selected to reach a consensus morphology (CM). Cases were classified into PB and INT subtypes using morphologic criteria given by Albores-Saavedra et al. [3]. Each pathologist documented the morphologic criteria present and classified them based on pure morphology.

Histomorphological criteria for classifying PAC into subtypes based on Albores-Saavedra

Intestinal-Type Adenocarcinomas

They resemble typical colonic adenocarcinoma with tubular/cribriform glands, tall columnar cells, dark, elongated, pseudostratified nuclei, and dirty luminal necrosis [3].

Pancreatobiliary Adenocarcinoma

They resemble pancreatic ductal carcinoma or cholangiocarcinoma with simple glands or papillary/ micropapillary architecture, cuboidal to low columnar cells, rounded nuclei, prominent nuclear atypia, and desmoplastic stroma [3].

In cases with both pancreatobiliary and intestinal type morphology, the presence of the predominant pattern (>50%) was considered for subtyping. The cases of consensus and discrepancy were noted. The H&E slides of the discrepant cases were reviewed in a multi-header microscope for consensus diagnosis, which was documented as CM. This was followed by interpretation of IHC markers.

Immunohistochemical staining and analysis

The corresponding sections from the paraffin block with viable tumor were chosen for IHC, and CK7, CK20, and CDX2 immunostaining were performed on deparaffinized sections using a two-step indirect method. Prediluted, ready-to-use CK7 (clone: OV-TL12/30, dilution: 1:200), CK20 (clone: Ks 20.8, dilution: 1:200), and CDX2 (clone: QR045, dilution: 1:200), Quartett (Bio CYC), antibody Germany were used. Positive membranous or cytoplasmic staining in at least 10% of tumor cells was considered positive CK7 and CK20 expression [5]. Nuclear staining for CDX2 in at least 10% of tumor cells was taken as positive [5]. Positivity of CK7 was taken as pancreatobiliary, while CK20 and CDX2 were taken as intestinal subtype. Overlapping staining patterns were taken as mixed, and all markers being negative was taken as inconclusive. After correlating IHC results with CM, a final "immunomorphological subtype" (IMS) was obtained.

Statistical analysis

Quantitative information was presented as range, mean, and standard deviation. Cohen’s Kappa statistics were used to assess the mutual agreement between the two pathologists. Kappa was also used to assess the agreement between the subtyping done by individual pathologists with the CM and between the CM and IMS. For comparative analysis between three subtypes, the chi-square test was applied. For survival analysis, the Kaplan-Meier test was applied to assess the association between subtypes and survival status. Statistical analysis was performed using standard statistical software, IBM SPSS Statistics for Windows (version 26.0, IBM Corp., Armonk, NY, 2019). A p-value of ≤0.05 was considered statistically significant.

## Results

A total of 33 pancreaticoduodenectomy specimens were found to satisfy the inclusion and exclusion criteria, morphologically suggestive of adenocarcinoma, and were received during the mentioned period. These were either from the CBD or periampullary duodenal mucosa. The cases were evaluated by two observers.

Slight agreement was noted in a few morphological features, like architecture (k = 0.186), presence of goblet cells (k = 0.138), luminal dirty necrosis (k = 0.154), and stromal desmoplasia (k = 0.148). No agreement was noted in the other morphological features, like cytomorphology (k = -0.168) and nuclear features (k = -0.222) (Table 1).

**Table 1:** Summary of the comparison of histomorphological features by two observers (n = 33) (as per the criteria proposed by Albores-Saavedra et al.) [3] n: number of cases; Obs: observer; SE of Kappa: standard error of the Kappa coefficient; CI: confidence interval

Sl No	Histomorphologic parameter	Obs 1 (n = 33) (%)	Obs 2 (n = 33) (%)	Kappa	SE of Kappa	95% CI	Agreement
1.	Architecture:						
	Simple branching/papillary	13 (39.4%)	14 (42.4%)	0.186	0.172	-0.152 to 0.524	Slight
	Tubular/cribriform/ solid nest	20 (60.6%)	19 (57.6%)				
2.	Cytomorphology:						
	Cuboidal to columnar	17 (51.5%)	24 (72.7%)	-0.168	0.154	-0.469 to 0.134	No
	Tall columnar	16 (48.5%)	09 (27.3%)				
3.	Nucleus:						
	Round/single layer	14 (42.4%)	21 (63.6%)	-0.222	0.160	-0.535 to 0.091	No
	Oval/elongated/pseudostratified	19 (57.6%)	12 (36.4%)				
4.	Goblet cell:						
	Absent	17 (51.5%)	25 (75.8%)	0.138	0.151	-0.157 to 0.434	Slight
	Present	16 (48.5%)	08 (24.2%)				
5.	Luminal dirty necrosis:						
	Absent	15 (45.5%)	17 (51.5%)	0.154	0.171	-0.181 to 0.488	Slight
	Present	18 (54.5%)	16 (48.5%)				
6.	Desmoplastic stroma:						
	Present	17 (51.5%)	19 (57.6%)	0.148	0.171	-0.188 to 0.483	Slight
	Absent	16 (48.5%)	14 (42.4%)				

Histomorphological subtyping

Observer 1 identified the INT subtype in 60.6% (20/33) cases and PB in 39.4% (13/33) cases. Observer 2 identified the INT subtype in 27.3% (09/33) cases, PB in 69.7% (23/33) cases, and 03% (01/33) cases of mixed subtype. Upon review, based on the CM, there were 33.3% (11/33) cases in INT subtype and 66.7% (22/33) in PB subtype (Table 2).

**Table 2:** Distribution of PAC cases by two different observers and on basis of consensus morphology (n = 33) INT: intestinal; PB: pancreatobiliary; CM: consensus morphology

Morphological type	Obs 1 (n = 33) (%)	Obs 2 (n = 33) (%)	CM (n) (%)
INT	20 (60.6)	09 (27.3)	11 (33.3)
PB	13 (39.4)	23 (69.7)	22 (66.7)
Mixed	00 (0.0)	01 (03.0)	00 (0.0)
Total	33 (100.0)	33 (100.0)	33 (100.0)

Interobserver agreement of histological classification

On comparing the subtypes of the independent slide assessment between both observers, a concordance was noted in 16 cases out of 33 cases (48.5%). Among these 16 cases, 6/33 cases were of INT subtype and 10/33 cases were of PB subtype. There was no agreement between observer 1 and observer 2 (k = 0.08). This result was not statistically significant (p = 0.622) (Table 3).

**Table 3:** Agreement between the two pathologists (n = 33) MIX: mixed

			Observer 1 (n = 33)		Kappa	p-value
			INT (n = 20)	PB (n = 13)		
Observer 2 (n = 33)	INT (n = 09)	Count	6	3	0.08	0.622
		% within Obs 1	30.0%	23.1%		
	MIX (n = 01)	Count	1	0		
		% within Obs 1	5.0%	0.0%		
	PB (n = 23)	Count	13	10		
		% within Obs 1	65.0%	76.9%		

**Figure 1:**
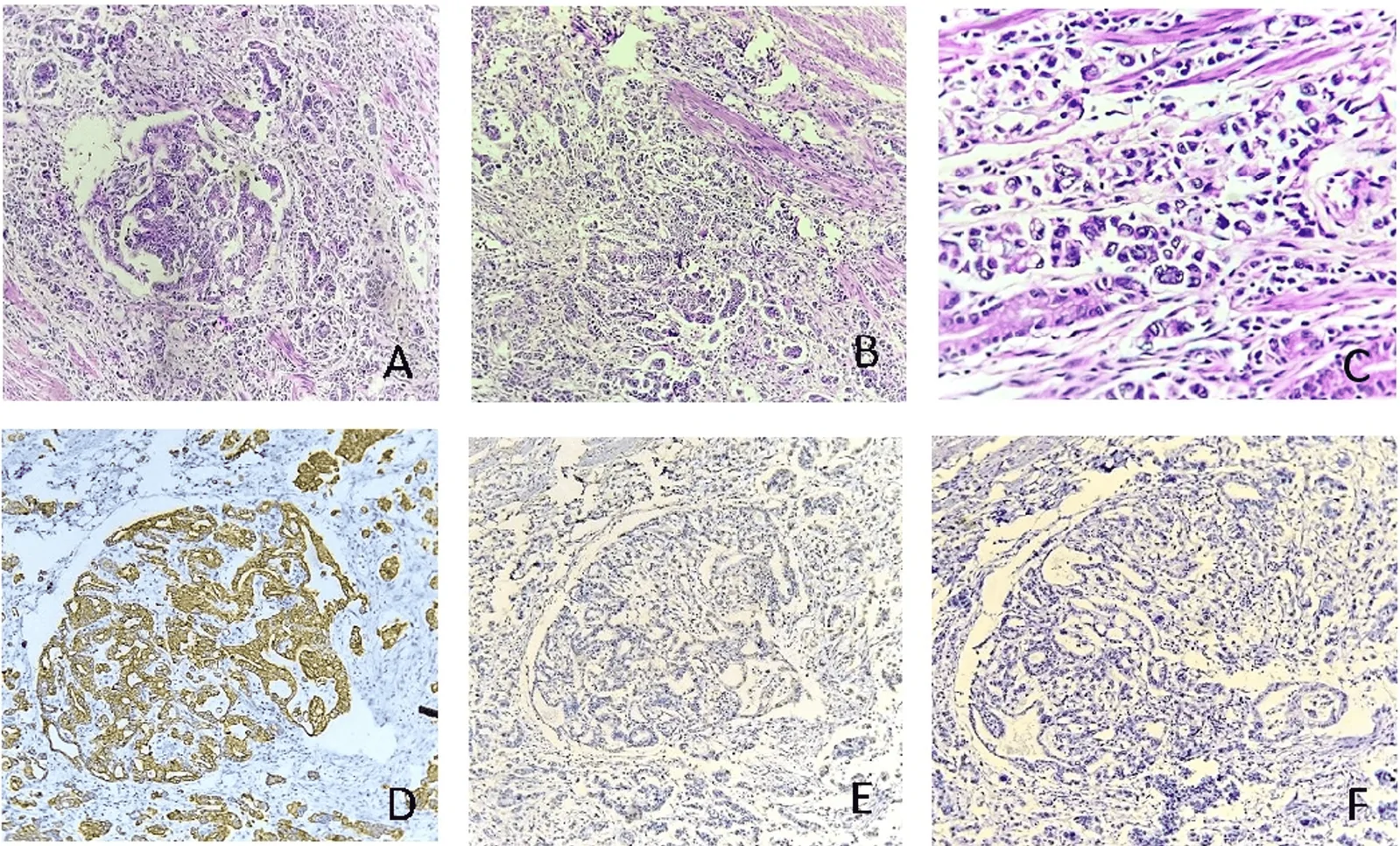
A case of immunomorphologically PB subtype with interobserver discrepancy showing overlapping features (A, B: 100x). Histomorphology showing cribriform pattern (A), simple tubular glands (B), with poor differentiation (C) (C: 400x) (A-C: H&E). IHC showing (D) CK7 membranous-positive, (E) CK20-negative, (F) CDX2-negative (D-F: IHC, 100x). PB: pancreatobiliary, H&E: hematoxylin and eosin, IHC: immunohistochemistry, CK7: cytokeratin 7, CK20: cytokeratin 20, CDX2: caudal type homeobox 2

**Figure 2:**
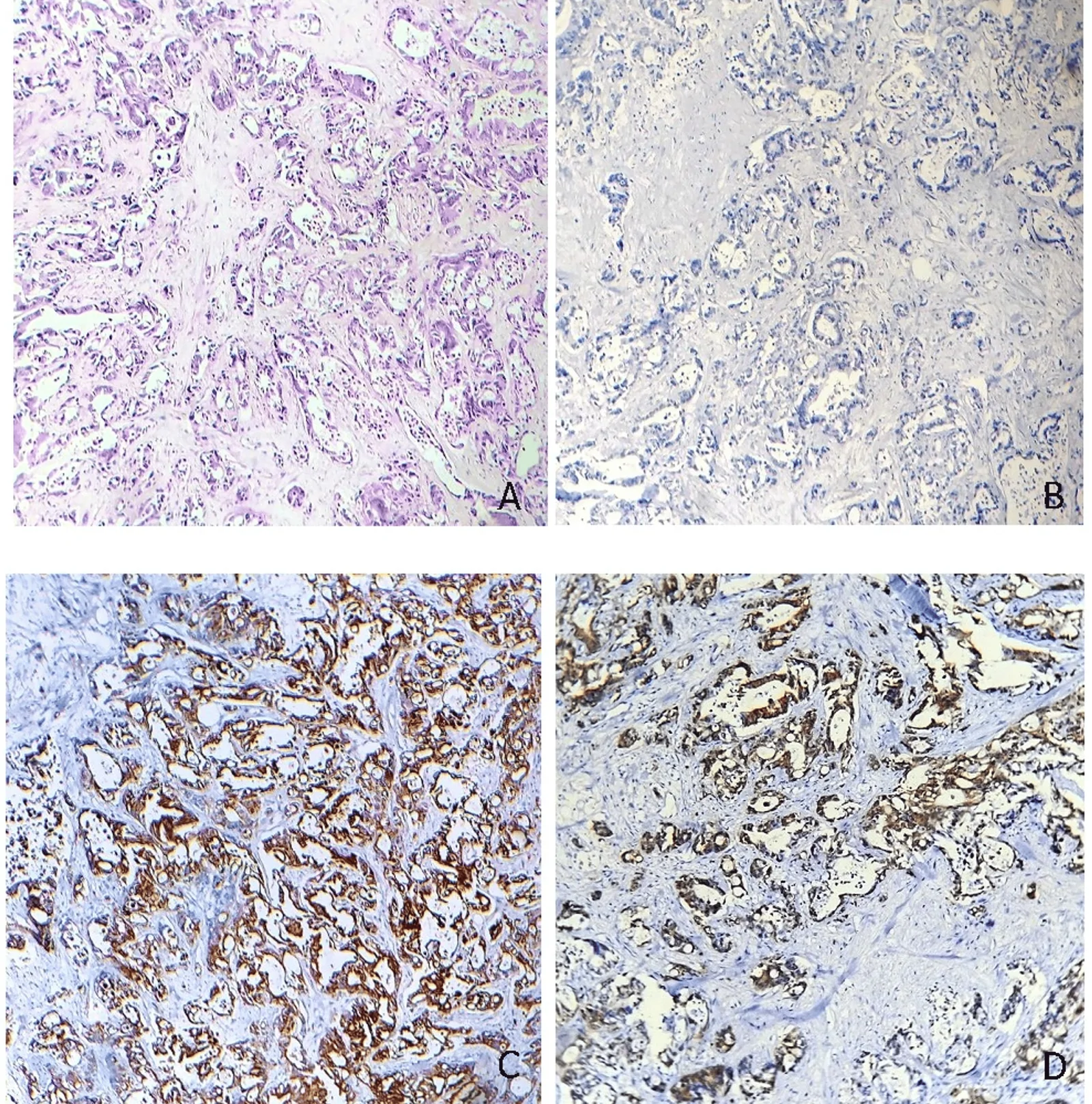
A case of immunomorphologically INT subtype mimicking PB. (A) Tubular glands embedded in desmoplastic stroma. (B) CK7-, (C) CK20+, (D) CDX2+ (A-D: 100x; A: H&E; B-D: IHC). INT: intestinal; PB: pancreatobiliary

Few cases of PB subtype, which showed interobserver variation upon review, revealed cases with poor differentiation, poor preservation, or extensive goblet cells (Figure 1). On reviewing cases of INT subtype with interobserver variation, cases showed areas of extensive desmoplastic stroma (Figure 2).

Fair agreement (k = 0.151) was noted between observer 1 and CM, which was not statistically significant (p = 0.314). Moderate agreement (k = 0.455) was noted between observer 2 and CM, which was statistically significant (p = 0.010) (Table 4).

**Table 4:** Agreement between both observers and consensus for morphology (n = 33)

			CM		Kappa	p-value
			INT	PB		
Observer 1 (n = 33)	INT (n = 20)	Count	8	12	0.151	0.314
		% within CM	72.7%	54.5%		
	PB (n = 13)	Count	3	10		
		% within CM	27.3%	45.5%		
Observer 2 (n = 33)	INT (n = 09)	Count	6	3	0.455	0.010
		% within CM	54.5%	13.6%		
	MIX (n = 01)	Count	1	0		
		% within CM	9.1%	0.0%		
	PB (n = 23)	Count	4	19		
		% within CM	36.4%	86.4%		

There was a moderate degree of agreement (k = 0.588, p < 0.001) between CM and IMS, which suggested that the agreement between morphological subtype based on consensus of two pathologists and immunomorphological subtype was statistically significant (Table 5).

**Table 5:** Agreement between consensus for morphology and immunomorphological subtype (n = 33) MIX: mixed

		CM (n = 33)		Kappa	p-value
		INT (n = 11)	PB (n = 22)		
Immunomorphological subtype (IMS) (n = 33)	INT (n = 08)	8	0	0.588	<0.001
	MIX (n = 05)	1	4		
	PB (n = 20)	2	18		

The PB subtype was found in younger age groups, whereas the INT subtype was noted only in older age groups. No statistically significant correlation was identified between IMS and age (p = 0.418), gender (0.302), grade (0.900), LVI (0.193), PNI (0.452), pT (0.060), pN (p = 0.659), and stage (p = 0.519) (Table 6).

**Table 6:** Comparison between immunomorphological subtype with various clinicopathological parameters (n = 33) IMS: immunomorphological subtype, LVI: lymphovascular invasion, PNI: perineural invasion, pT: pathological primary tumor, pN: pathological node

		IMS			p-value
		PB	INT	MIX	
Age (y)	31-40 (n = 03)	3 (15.0%)	0 (00.0%)	0 (00.0%)	0.418
	41-50 (n = 06)	1 (05.0%)	3 (37.5%)	2 (40.0%)	
	51-60 (n = 13)	8 (40.0%)	3 (37.5%)	2 (40.0%)	
	61-70 (n = 08)	6 (30.0%)	1 (12.5%)	1 (20.0%)	
	71-80 (n = 03)	2 (10.0%)	1 (12.5%)	0 (00.0%)	
Sex	F (n = 14)	8 (40.0%)	5 (62.5%)	1 (20.0%)	0.302
	M (n = 19)	12 (60.0%)	3 (37.5%)	4 (80.0%)	
Grade (G)	G 1 (n = 24)	15 (75.0%)	5 (62.5%)	4 (80.0%)	0.900
	G 2 (n = 06)	3 (15.0%)	2 (25.0%)	1 (20.0%)	
	G 3 (n = 03)	2 (10.0%)	1 (12.5%)	0 (00.0%)	
LVI	- (n = 21)	14 (70.0%)	3 (37.5%)	4 (80.0%)	0.193
	+ (n = 12)	6 (30.0%)	5 (62.5%)	1 (20.0%)	
PNI	- (n = 26)	15 (75.0%)	6 (75.0%)	5 (100.0%)	0.452
	+ (n = 07)	5 (25.0%)	2 (25.0%)	0 (00.0%)	
pT	pT1 (n = 01)	0 (00.0%)	0 (00.0%)	1 (20.0%)	0.060
	pT2 (n = 19)	11 (55.0%)	5 (62.5%)	3 (60.0%)	
	pT3 (n = 13)	9 (45.0%)	3 (37.5%)	1 (20.0%)	
pN	pN0 (n = 25)	15 (75.0%)	5 (62.5%)	5 (100.0%)	0.659
	pN1 (n = 05)	3 (15.0%)	2 (25.0%)	0 (00.0%)	
	pN2 (n = 03)	2 (10.0%)	1 (12.5%)	0 (00.0%)	
Stage	Ⅰ (n = 16)	9 (45.0%)	3 (37.5%)	4 (80.0%)	0.519
	Ⅱ (n = 09)	6 (30.0%)	2 (25.0%)	1 (20.0%)	
	Ⅲ (n = 08)	5 (25.0%)	3 (37.5%)	0 (00.0%)	

**Figure 3:**
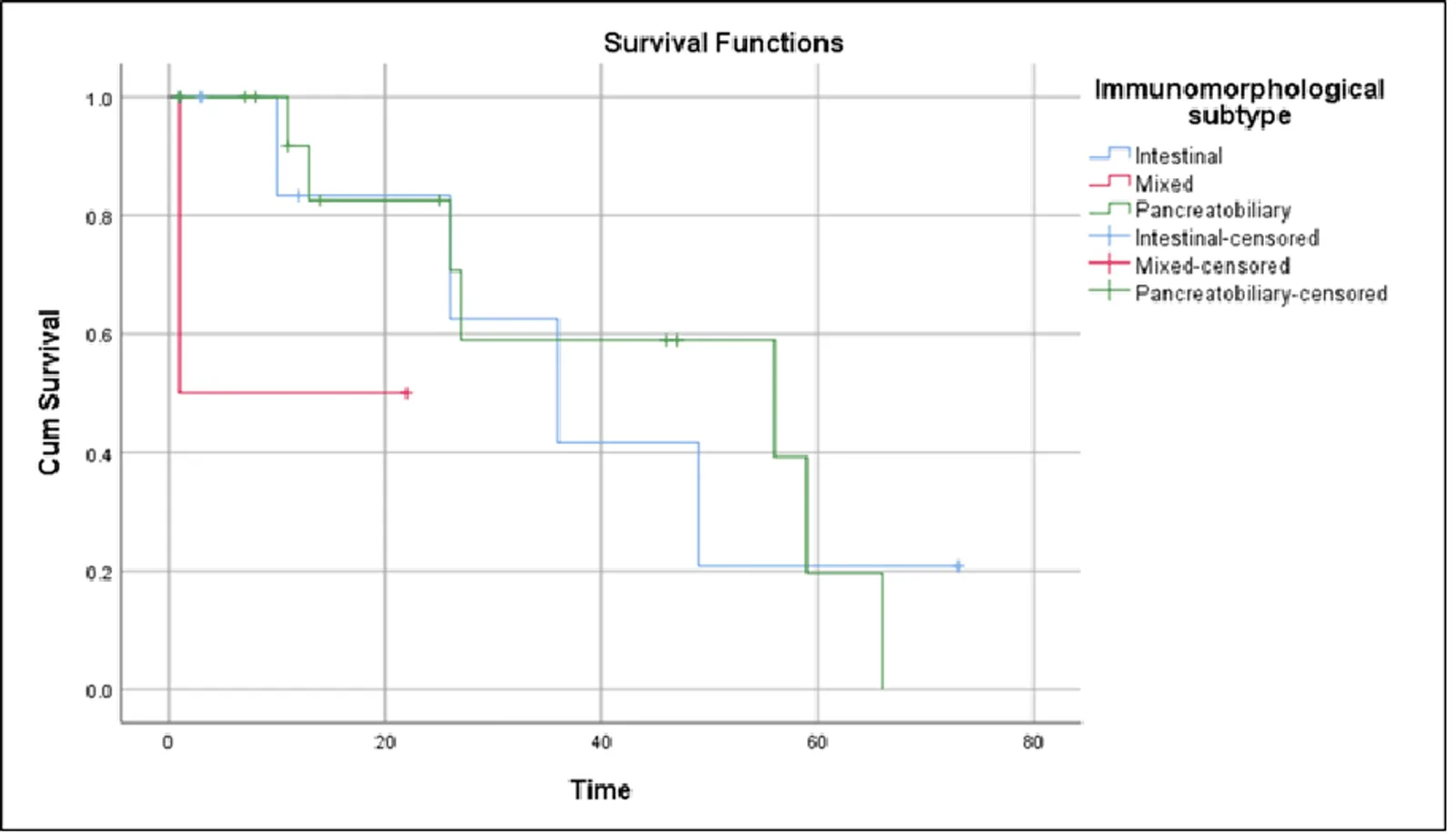
Kaplan-Meier survival curve showing association of immunomorphological subtype (IMS) with survival status. Immunomorphological subtype: blue line – intestinal, red line – mixed, green line – pancreatobiliary, blue tick (+) – intestinal (censored), red tick (+) – mixed (censored), green tick (+) – pancreatobiliary (censored)

Kaplan-Meier survival analysis demonstrated differences in survival patterns among immunomorphological subtypes. The MIX subtype showed an early decline in survival, while the INT subtype demonstrated a gradual reduction over time. The PB subtype maintained better survival initially but showed a later decline. However, it was not statistically significant (p = 0.311) (Figure 3). 

## Discussion

PAC is an uncommon malignant tumor involving an anatomically complex site, which shows two common histomorphological subtypes, namely, "pancreatobiliary," which replicates the lining epithelium of the pancreatic duct, and "intestinal," which resembles colonic-type intestinal epithelium [1,6]. This study was undertaken to understand the importance of a detailed histomorphology in subtyping of PAC and the role of IHC as an adjunct to its subtyping. This categorization holds therapeutic and prognostic significance, as the former is treated with the gemcitabine-based chemotherapy regimen and has poorer survival, whereas the latter is treated with 5-fluorouracil chemotherapy and has comparatively better survival [7]. Our study was distinct in its objective of evaluating interobserver variability in the histomorphology of PAC.

In the present study, a total of 33 pancreaticoduodenectomy specimens were studied by two different pathologists for histomorphological subtyping, followed by interobserver variation assessment and CM. Correlating the histomorphology and IHC (CK7, CK20, and CDX2), an IMS was made. Histomorphological features proposed by Albores-Saavedra have been used, but the usefulness of these histomorphological features has not been evaluated by authors. In this study, we have highlighted the individual diagnostic criteria that have been included by authors, including Kwon et al. [4]. Interobserver comparison on histomorphological parameters in the present study showed that architecture (k = 0.186), desmoplastic stroma (k = 0.148), necrosis (k = 0.154), and presence of goblet cells (k = 0.138) had slight agreement, whereas the cytologic (k = -0.168) and nuclear morphology (k = -0.222) did not show good agreement. This has not been highlighted by most authors who have done similar studies. This lack of reproducibility of the morphologic parameters contributes to the interobserver variation. The interobserver variation was poor in the present study (k = 0.08), but it was not statistically significant (p = 0.622). The minimum essential histomorphologic parameters, the number of sections, and the essential field areas required to arrive at a specific subtype have not been specified by any authors previously. Also, in cases with the presence of more than one criterion showing overlapping features, there are no specific guidelines available till now. This leads to an increase in interobserver variation and compromises reproducibility. This difference in the morphologic subtyping could be resolved only with a consensus review of the slides as shown in the present study.

Compared to the present study, the interobserver variation was found to be better in a study done by Kwon et al. [4]. However, they also had cases with interobserver variation, likely because the ampullary region is a transition area between intestinal and pancreatobiliary zones [4]. Furthermore, histomorphology is useful, but there is no definite gold standard for reference [4]. We had cases with intestinal-type cytoarchitectural morphology surrounded by dense desmoplastic stroma, which turned out to be the INT subtype in the final IMS diagnosis. However, the desmoplasia led to misclassification as the PB subtype on morphology alone. On the contrary, we also had cases of mixed types showing pancreatobiliary pattern and extensive goblet cells, causing discrepancy between the observers. As there is no specific guideline as to how many features are essential to subtype using the Albores-Saavedra [3]. Tumor heterogeneity, slide-to-slide variation, and different areas of examination by pathologists with variable experience can account for this [8]. Tanager et al., similar to us, also found that, in many cases where concordance was lacking, it was of mixed type, possibly from the junction between the intestinal and pancreatobiliary epithelium [8]. It could also be because of divergent differentiation as a part of tumor progression. Mixed types often contributed to the failure of agreement between observers. Akin to the findings of this study, Ang et al. found that overlapping features of both subtypes can occur [9]. Similar to our study, they also observed discrepancies in subtyping of these tumors, which were sorted after reviewing the slides together again [9].

In our experience, another reason for this variation was acknowledging only the predominant pattern, which was also done by Reid et al [10]. This requires examining the tumor area in multiple slides to ensure adequate representation of all the subtypes [10]. Interestingly, Reid et al. subtyped tumors by using both approaches, i.e., "purist" and "predominant" [10]. Their agreement was comparable to the present study (k = 0.44) with the use of predominant subtype [10]. The mixed-subtype cases show a transition from pancreatobiliary-type to intestinal-type epithelium because the former is present mostly toward the invasive focus, which can be easily missed if we only focus on the predominant pattern [10]. The mixed pattern of IHC markers likely represents true tumor heterogeneity.

In our study, no significant agreement (k = 0.08) was seen between the two observers, as only 48.5% of cases showed concordance using pure morphology. First, periampullary carcinomas are often of hybrid phenotypes, which leads to considerable subjectivity [10]. Second, phenotypic variations are also noted depending on the extent of invasion of the tumor [11]. Third, as tumor cells take origin from a site where both PB- and INT-type histologies coexist, morphological disagreements are common as this is a site of confluence [8]. Fourth, subtyping was done following the "predominant pattern" approach in the present study. Hence, the minor subtypes were missed, similar to Reid et al., who found that the binary system can actually pose problems in the histomorphologic subtyping [10]. Furthermore, a host of factors distort the periampullary architecture, ranging from infiltration by malignant cells, inflammation, or even fibrosis, which is further complicated by anatomical variations seen from person to person [10,12]. These parameters were also addressed by Reid et al. and Sarmiento et al. as contributing factors for discrepancies [10,12]. Lastly, the poor differentiation shown by high-grade tumors causes difficulties in subtyping due to poorly appreciated histomorphology, which also leads to interobserver variation, as was also observed by Bakshi et al. [7]. All these factors can contribute to difficulty in subtyping based on pure histomorphology. Hence, extensive sampling is essential to ensure the optimal assessment of major and minor areas, which will increase the reproducibility and thereby minimize the interobserver variation.

Because of the above-mentioned reasons leading to interobserver variation, a CM was done on a multi-header microscope in the present study. CM showed fair agreement with the first observer (k = 0.151), but a moderate degree of agreement was achieved with the second observer, which was statistically significant too (k = 0.455, p = 0.010). This indicates that in cases of discrepancy, the use of CM is essential to increase the agreement, which aligns with the studies done by Tanager et al. and Ang et al. [8,9].

This study is unique in addressing PAC, which is an uncommon malignancy, particularly as it addresses the individual histomorphologic parameters. Integration of morphology with IHC markers and use of immunomorphological diagnosis (IMS) are other merits of the study.

There were certain limitations of this study, which were limited sample size, no comparison with the "purist" approach as only the "predominant" approach was used, no use of apomucins, and a lack of follow-up data for all cases.

## Conclusions

Therefore, based on this study, we can conclude that there will remain inevitable discrepancies in interobserver variations in the histomorphology-based subtyping of periampullary tumors, which can be minimized by optimal sampling and use of CM. This study has thrown light on the usefulness of IHC as an adjunct to morphology in the subtyping of these tumors, with CK7 being useful for pancreatobiliary subtype while CK20 and CDX2 being beneficial in identifying the intestinal subtype. With optimal sampling, use of CM along with adjunct immunohistochemistry to recognize the immunomorphologic subtype, accurate classification of PAC is possible in most cases, which has therapeutic and prognostic significance.
